# Electrospun Scaffold Micro-Architecture Induces an Activated Transcriptional Phenotype within Tendon Fibroblasts

**DOI:** 10.3389/fbioe.2021.795748

**Published:** 2022-01-12

**Authors:** Mathew J. Baldwin, Jolet Y. Mimpen, Adam P. Cribbs, Edward Stace, Martin Philpott, Stephanie G. Dakin, Andrew J. Carr, Sarah JB. Snelling

**Affiliations:** Nuffield Department of Orthopaedics, Rheumatology and Musculoskeletal Sciences (NDORMS), University of Oxford, Oxford, United Kingdom

**Keywords:** tendon, biomaterial, fibroblast activation, electrospinning, rotator cuff augmentation

## Abstract

Biomaterial augmentation of surgically repaired rotator cuff tendon tears aims to improve the high failure rates (∼40%) of traditional repairs. Biomaterials that can alter cellular phenotypes through the provision of microscale topographical cues are now under development. We aimed to systematically evaluate the effect of topographic architecture on the cellular phenotype of fibroblasts from healthy and diseased tendons. Electrospun polydioxanone scaffolds with fiber diameters ranging from 300 to 4000 nm, in either a highly aligned or random configuration, were produced. Healthy tendon fibroblasts cultured for 7 days on scaffolds with highly aligned fibers demonstrated a distinctive elongated morphology, whilst those cultured on randomly configured fibers demonstrated a flattened and spread morphology. The effect of scaffold micro-architecture on the transcriptome of both healthy and diseased tendon fibroblasts was assessed with bulk RNA-seq. Both healthy (*n* = 3) and diseased tendon cells (*n* = 3) demonstrated a similar transcriptional response to architectural variants. Gene set enrichment analysis revealed that large diameter (≥2000 nm) aligned scaffolds induced an upregulation of genes involved in cellular replication and a downregulation of genes defining inflammatory responses and cell adhesion. Similarly, *PDPN* and *CD248*, markers of inflammatory or “activated” fibroblasts, were downregulated during culture of both healthy and diseased fibroblasts on aligned scaffolds with large (≥2000 nm) fiber diameters. In conclusion scaffold architectures resembling that of disordered type III collagen, typically present during the earlier phases of wound healing, resulted in tendon fibroblast activation. Conversely, scaffolds mimicking aligned diameter collagen I fibrils, present during tissue remodelling, did not activate tendon derived fibroblasts. This has implications for the design of scaffolds used during rotator cuff repair augmentation.

## Introduction

Shoulder pain is the third most common cause of musculoskeletal pain, with an estimated prevalence of 16–26% in the United Kingdom. (Urwin et al., 1998). Rotator cuff tendon tears constitute a large proportion of this disease burden, resulting in pain, disability and a significant socioeconomic cost. (Mitchell et al., 2005; Piitulainen et al., 2012; Judge et al., 2014). Indeed, tears of the rotator cuff affect around 50% of those over 66 years of age. (Yamaguchi et al., 2006). Unfortunately, tendon tears demonstrate little capacity for endogenous repair with surgical intervention frequently required. Over 10,000 rotator cuff repairs are performed annually in the United Kingdom and over 200,000 in the United States. (Judge et al., 2014). While resolution of symptoms correlates with rotator cuff healing (Carr et al., 2017), current surgical techniques result in functionally incompetent scar tissue with over 40% of rotator cuff repairs failing within 2 years of surgery. (Carr et al., 2017). In an attempt to improve tendon healing rates, biomaterial patches that overlay tendon tears and provide cell instructive signals alongside mechanical support, are under development. (Mouthuy et al., 2014; Hakimi et al., 2015).

The nanoscale structure of the extracellular matrix (ECM) provides a natural network of physical and chemical cues that supports cells and guides their behaviour. (Crowder et al., 2016). As the primary constituent of tendon ECM, collagen fibers, which vary in diameter (100–4000 nm) (Wang, 2006) and orientation, are a critical determinant of a tendon fibroblasts’ (the predominant cell type in tendon) physical and mechanical environment. Tendon tears alter this critical ECM niche. (Riley et al., 1994). Diseased tendon exhibits an overall reduction in collagen content, a relative increase in collagen II and III and a loss of collagen alignment. (Dean et al., 2012). In turn, these changes may contribute to the phenotypic shift that diseased tendon fibroblasts undergo. (Chaudhury et al., 2016). Tendon fibroblasts isolated from rotator cuff tears exhibit altered collagen synthesis and a constitutive pro-inflammatory profile–a phenotype that persists despite multiple rounds of cell culture. (Dakin et al., 2015, 2017a; Kendal et al., 2017). Indeed, persistent fibroblast activation is a hallmark feature of many chronic fibrotic and inflammatory conditions (Filer et al., 2015), and describes a diverse cellular response that includes a flattened morphology through cytoskeletal alterations, proliferation, synthesis of extracellular matrix, cytokine secretion and recruitment of immune cells.

The surgical implantation of electrospun scaffolds provides cells with a synthetic extracellular niche. Modifying a synthetic biomaterial’s microarchitecture can therefore recapitulate a “healthy” biophysical environment, and in doing so has the potential to redirect diseased tendon fibroblasts toward a healthy phenotype. Indeed, diseased rotator cuff fibroblasts demonstrate higher rates of proliferation, collagen and glycosaminoglycans production when cultured on aligned electrospun polydioxanone (PDO) scaffolds with submicron fiber diameters. (Erisken et al., 2013).

However, previous studies have focused on a limited number of architectural modifications of synthetic biomaterials and have not considered their global effect on cellular phenotype, using only a small repertoire of genes or surface proteins to define a favourable response. Consequently, the ideal microarchitectural properties of synthetic scaffolds has remained elusive. Our aim was to robustly interrogate a broad range of microarchitectural properties, assessing their effect on the global transcriptional profiles of fibroblasts isolated from both healthy and diseased tendons.

## Methods

### Patient and Public Involvement

Patients are actively involved during biomaterial implant development meetings held at the NIHR Biomedical Research Centre in Oxford.

### Electrospun Scaffold Fabrication

#### Preparation of the Electrospinning Solution

The electrospinning solutions were prepared by dissolving variable quantities of PDO resin (Riverpoint Medical, Portland, United States) in Hexafluoro-2-propanol (Apollo Scientific Ltd, Cheshire, United Kingdom), with or without the addition of pyridine (purity >99.5%, PhEur grade, EMSURE ACS, Merck, Darmstadt, Germany) (Lach et al., 2019), to obtain the required mass concentration (% w/v) of PDO solution (Supplementary Tables S1, S2). The solution was agitated at room temperature on a roller set on speed of 15 RPM for at least 24 h to allow for complete dissolution of the polymer.

#### Production of ES Scaffolds

PDO Solutions were loaded into a syringe pump (Harvard Apparatus–PHD 2000; Kent, United Kingdom) and supplied to an environmentally-controlled (20^o^C and 30% humidity) IME electrospinning device (IME Technologies, Spaarpot, Netherlands) at a constant flowrate. A voltage was then applied between a stainless-steel nozzle and rotating collector coated in a single sheet of aluminium foil. Variations of; flowrate, voltage, collector speed and nozzle-collector distance, enabled the production of scaffolds with different fiber diameters and alignments. The final parameters utilised in the production of scaffolds for *in vitro* culture are outlined in Supplementary Tables S1 and 2. Following production, scaffolds were stored in a desiccator to slow hydrolytic breakdown and utilised within 30 days.

#### Scanning Electron Microscopy and Image Analysis

A 1 cm^2^ section of ES scaffold was mounted on an aluminium stub using a carbon adhesive disk and gold-coated using a sputter coater (SC7620 mini, Quantum Design, Switzerland). High-resolution images were taken using an environmental scanning electron microscope (Evo LS15 Variable Pressure Scanning Electron Microscope, Carl Zeiss, Germany). Multiple areas (minimum of 3) were observed randomly and the microfiber diameter was measured based on images taken at ×5,000 magnification using ImageJ (National Institute of Healthy, Maryland, United States). (Rueden et al., 2017). A total of 20 measurements were performed for type of sample. Scaffold pore sizes were quantified from SEM image analysis in ImageJ. SEM images were first segmented to produce a binary black and white image. This was produced through global thresholding, in which a single constant threshold was applied. All pixels greater than or equal to the threshold were defined as electrospun fibres and the remaining belonged to the background. The optimal threshold differed between images and was chosen based on observer judgement. To improve segmentation quality, manual correction of each segmented image was then undertaken. The pore size was then estimated using the built-in “Analyse Particle” plugin in ImageJ. For each scaffold, this was undertaken for a minimum of five discrete images and values averaged.

#### Isolation of Primary Human Fibroblasts for *in vitro* Culture

Informed consent was provided by all donor and samples collected through Oxford Musculoskeletal Biobank (REC 09/H0606/11), in full compliance with national and institutional ethical requirements including the Declaration of Helsinki (2013) and the Human Tissue Act (2004). Biopsies of diseased rotator cuff tendons were collected from patients undergoing tendon repairs. All tendon tears had been classified as massive (>5 cm in anterior-posterior diameter) by the operative surgeon. Healthy controls were obtained from excess hamstring tendon collected from patients undergoing anterior cruciate ligament reconstruction using hamstring grafts. Both torn rotator cuff and hamstring tissue samples were cut into approximately 2 mm^2^ sections and transferred into 6-well plates (Corning Inc., Corning, NY, United States). Samples were initially cultured in D50 culture media (DMEM/F12 containing 50% FCS and 1% PS) in a tissue culture incubator at 37^o^C and 5% CO_2_. Phase contrast microscopy was used to determine the migration of fibroblasts out of tendon explants, at which point whole tissue samples were removed and the culture medium changed to D10 media. At 90% confluence cells were enzymatically detached (TrypLE™, ThermoFisher Scientific, Waltham, MA, United States) and cryopreserved. All subsequent experiments were undertaken on cells at passage 2 (*p* = 2) to avoid phenotypic drift experienced at higher passages. (Yao et al., 2006).

### Cell Culture for RNA Sequencing

#### Preparation of ES Scaffolds for *in vitro* Cell Culture

1.5 cm × 1.5 cm sections of scaffolds were cut and sprayed with 70% ethanol, facilitating detachment and sterilisation of the scaffold. Sections were then mounted onto 24-well sized cell crowns (Scaffdex, Tampere, Finland), two further rounds of ethanol sterilisation undertaken and rinsed five times with 1 x Phosphate Buffered Saline (PBS, Merck, Germany).

To facilitate protein adsorption and subsequent cell attachment, mounted electrospun scaffolds were placed in 24-well plates with 2 mls D10 culture media for 2 h. Scaffolds underwent a further three PBS wash cycles to remove non-adsorbed proteins and mounted.

#### Seeding and Culture of Tendon Fibroblasts

Tendon fibroblasts from healthy (*n* = 3) and diseased (*n* = 3) tendons were counted (TC-20 Automated Counter, Bio-Rad, Hertfordshire, United Kingdom) and 100 µL of cell suspension, equating to a seeding density of ∼40,000 cells/cm^2^, seeded directly on to the surface of each scaffold. Cells were left to adhere for 2 h prior to transfer of cell-laden scaffolds to 24-well plates (CostarⓇ, Corning Inc, Corning NY, United States) containing 1.5 ml of fresh D10 media. To ensure full immersion, an additional 500 µL of D10 media was then added to the upper surface of each scaffold. Media was changed every 3 days for the duration of each experiment. After 7 days, treatment medium was removed and scaffolds washed with 1x PBS, before being harvested in Trizol (Invitrogen, supplied by ThermoFisher), and stored at -80°C.

### Immunohistochemistry

Healthy tendon fibroblasts were cultured on scaffolds for 7 days before being fixed in 10% formalin for 10 min. Cells were then permeabilised with 0.1% Triton-X (ThermoFisher Scientific) for 3 min and washed in PBS. Specimens were then stained with AlexaFluor™ 488 Phalloidin (ThermoFisher Scientific) at 1 in 1000 concentration for 20 min at room temperature in the dark. Nuclear counter staining was performed with DAPI (ThermoFisher Scientific) at a concentration of 1 μg/ml for a further 10 min at room temperature in the dark. Scaffolds were subsequently washed and mounted onto adhesive glass slides using a hard setting fluorescent mounting medium (VectaShield Hardset, Vector Laboratories, CA, United States) and stored at 4°C until image acquisition.

Imaging was acquired with a confocal laser scanning microscope ZEISS LSM 880 (Carl Zeiss) with 63 x 1.4 NA oil objective and equipped with an Airyscan detection unit. For each scaffold a 12-part titled scan (Zen 2.0, Carl Zeiss) was performed with a 15 percent overlap between tiles. Image stitching was undertaken in ImageJ.

### Bulk RNA Sequencing

RNA was extracted using a Direct-zol MicroPrep kit with DNase treatment (Zymo Research, Irvine, CA, United States) following the manufacturer’s instructions. RNA concentration was measured using a NanoDrop spectrophotometer and RNA quality assessed using High Sensitivity RNA ScreenTapes (Agilent, Santa Clara, CA, United States) on an Agilent 2,200 TapeStation. All samples had a RIN score of 7 or greater and no evidence of DNA contamination. A total of 100 ng RNA per sample was used to generate sequencing libraries using the NEBNext Ultra II Directional RNA Library Prep Kit for Illumina with poly-A selection (Illumina, San Diego, CA, United States) following the manufacturer’s instructions. Libraries from 24 samples with unique identifiers were pooled and run on an Illumina NextSeq 500 using the 75 cycles NextSeq High Output kit (Illumina).

Raw FASTQ files containing reads were generated by the Illumina software CASAVA v1.8. The raw FASTQ files were processed using CGAT-fiow readqc and mapping workflows (https://github.com/cgat-developers/cgat-flow). (Cribbs et al., 2019) Downstream analyses were performed using R version 3.5.1 (R Foundation, Vienna, Austria), and RStudio version 1.1.456 (RStudio, Boston, MA, United States). Differential expression analysis was performed using the DESeq2 package (Love et al., 2014) using ‘apeglm’ method to apply the shrinkage of logarithmic fold change. (Zhu et al., 2019). The adjusted *p*-value (padj) and significance of changes in gene expression were determined by applying the Bonferroni-Hochberg correction of 5% false discovery rate. PCA plots were generated using the package ggplot2, heatmaps were generated using the package pheatmap (PCA and heatmaps in Supplementary Figures S2, S3), and EnhancedVolcano (Blighe et al., 2021) was used to create volcano plots. For heatmaps hierarchical clustering was performed on the top 500 PCA genes in the first three eigenvectors (PC1, PC3 and PC3) across all samples. As previously described, genes are clustered on the basis of Pearsons correlation. Samples are clustered on the basis of a Euclidian distance matrix with complete linkage. (Pollen et al., 2014). Geneset enrichment analysis for hallmark pathways was performed using the fgsea (Korotkevich et al., 2016) package on significantly upregulated genes (P_adj_ < 0.05) ranked by the sign of the fold change multiplied by the inverse log10 of the *p*-value. The Enrichment Map (Merico et al., 2010) plugin was used to visualise geneset networks in cytoscape (v.3.8.2) (Cline et al., 2007). Subcellular location of translated genes was retrieved using the UniprotR package (Soudy et al., 2020), visualised using the CellNetVis web interface (Heberle et al., 2017) and functionally annotated with the mygene package. (Xin et al., 2016).

### Statistical Analysis

#### ES Scaffold Fiber Diameter

The statistical analysis was performed with the GraphPad Prism software version 8 (GraphPad Software, San Diego, United States). Data in graphs were expressed as means with standard deviations. T-tests and standard ANOVA with Tukey’s multiple comparisons testing were used to examine statistical differences between groups. Results were considered significant for *p* < 0.05.

#### ES Scaffold Fiber Anisotropy

Analysis was performed with PAST3 v20 (Oyvind Hammer, Natural History Museum, University of Oslo). Circular arrow plots were generated and a Rayleigh’s R value calculated. (Cremers and Klugkist, 2018). A value of R = 1 indicates that all data shares the same directionality, whereas a value of R = 0 implies an even distribution of data around a circle. An arbitrary threshold of R > 0.9 was taken to mean that a scaffold had aligned fibers, and an R value of <0.3 was deemed to be a scaffold with randomly orientated fibers.

## Results

### Characterisation of Electrospun Scaffold Production for *in vitro* Cell Culture

**FIGURE 1:**
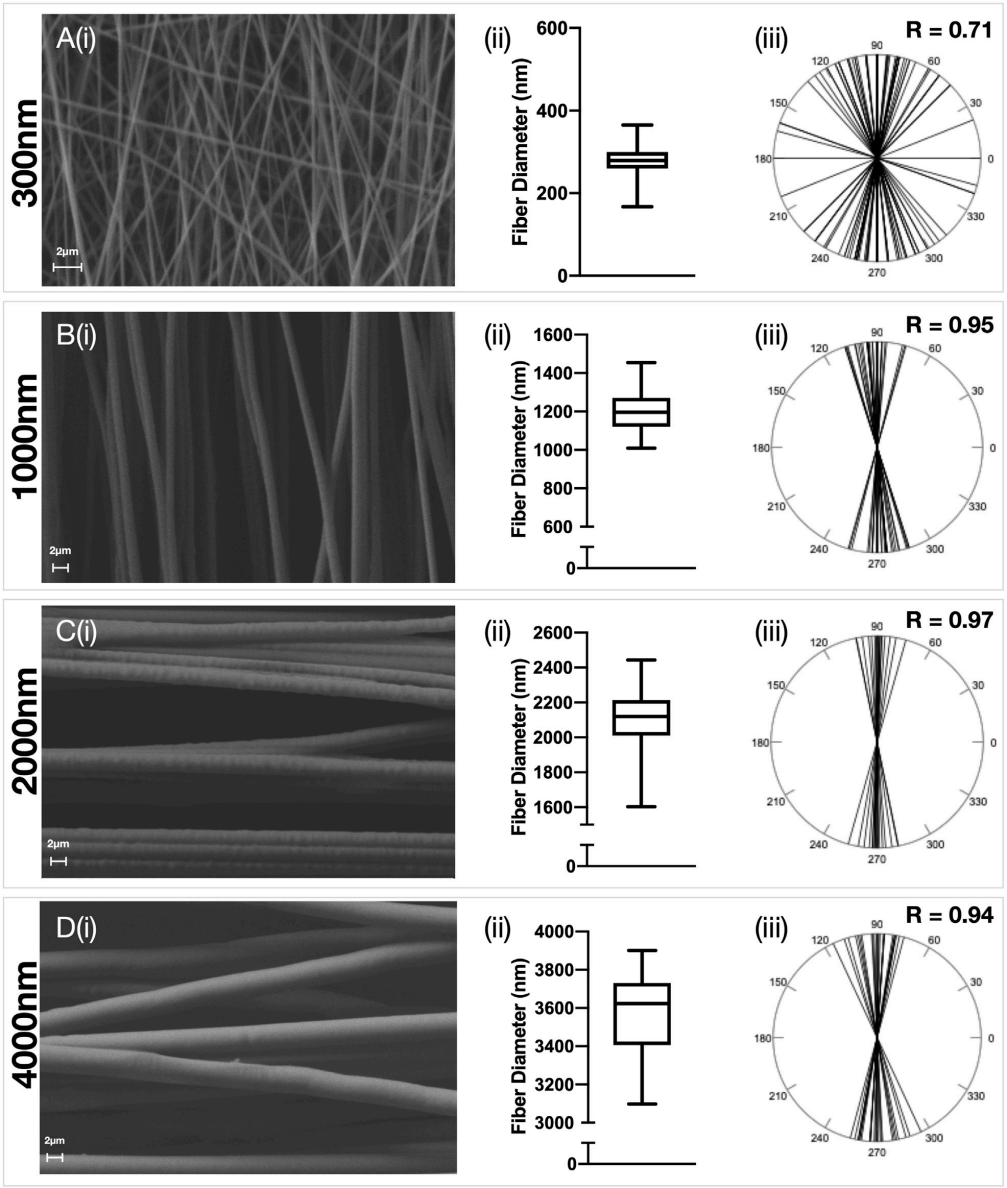
Characterisation of aligned electrospun fibers. Polydioxanone (PDO) fibers were produced in an aligned configuration and the fiber diameter manipulated to produce scaffolds with approximately **(A)** 300 nm **(B)** 1000 nm **(C)** 2000 nm and **(D**) 3,500 nm fibers. Each panel contains **(i)** Representative SEM image (5,000x–×10,000 magnification, scale bar = 2 µm) **(ii)** Fiber diameter distributions and **(iii)** Fiber alignment statistics.

**FIGURE 2:**
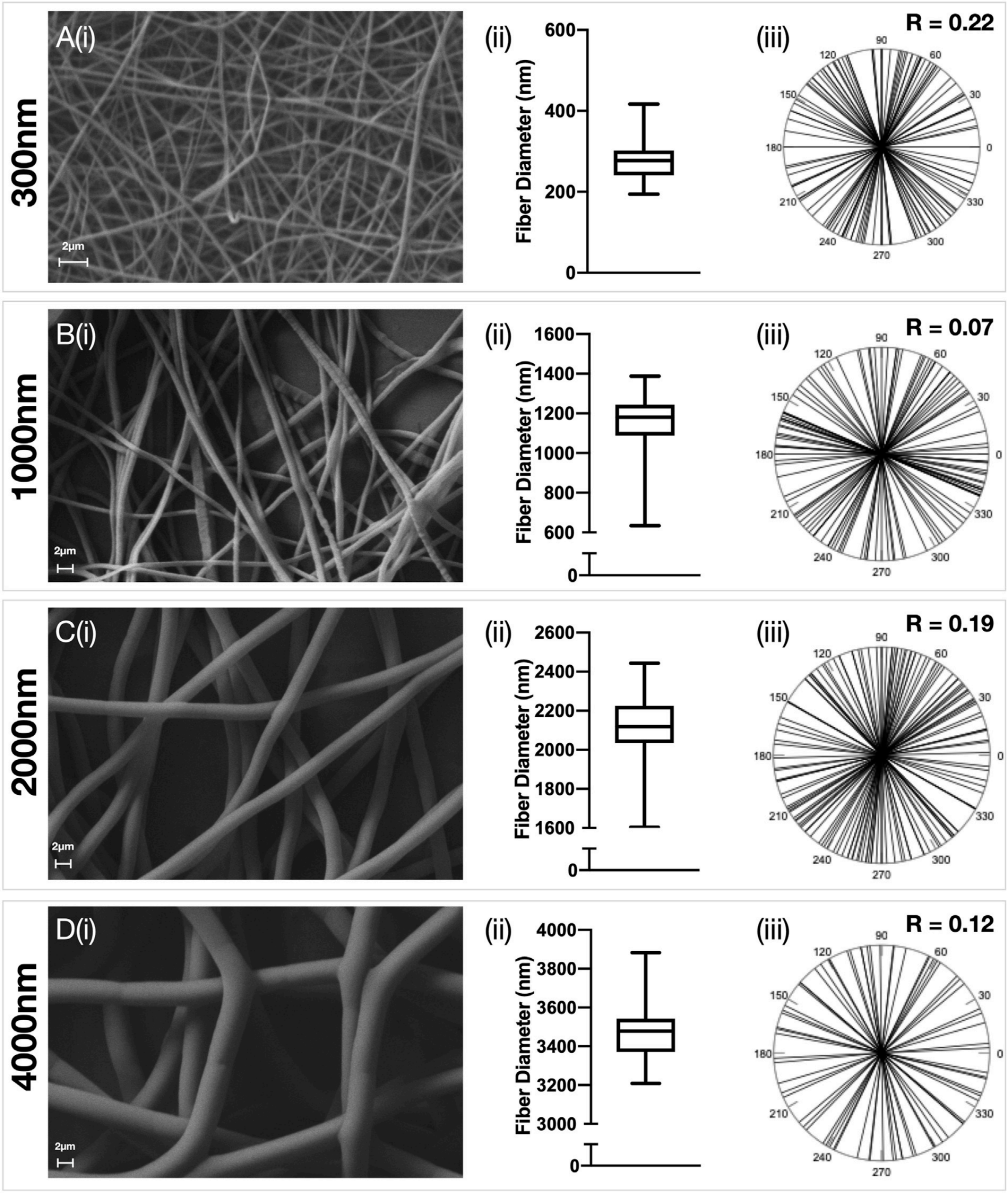
Characterisation of random electrospun fibers. Polydioxanone (PDO) fibers were produced in a random configuration and the fiber diameter manipulated to produce scaffolds with approximately **(A)** 300 nm **(B)** 1000 nm **(C)** 2000 nm and **(D)** 3500 nm fibers. Each panel contains **(i)** Representative SEM image (5,000x–×10,000 magnification, scale bar = 2 µm) **(ii)** Fiber diameter distributions and **(iii)** Fiber alignment statistics.

Electrospun PDO scaffolds with fibers in an aligned or random orientation were produced (Figures 1, 2 respectively) with a target mean fiber diameter of 300 nm, 1000 nm, 2000 nm or 4000 nm. There was no significant difference in the mean fiber diameter for scaffolds produced in an aligned or random orientation. A linear relationship between mean fiber diameter and mean pore size was observed for scaffolds with both aligned (R^2^ = 0.99, *p* = 0.0052) and randomly (R^2^ = 0.99, *p* = 0.0013) orientated fibers (Supplementary Figure S1). Aligned scaffolds with target fiber diameters of 1000 nm, 2000 and 4000 nm each demonstrated significantly larger pore sizes than their randomly orientated counterparts.

### Scaffold Micro-architecture Modulates Healthy Hamstring Fibroblast Morphology

**FIGURE 3:**
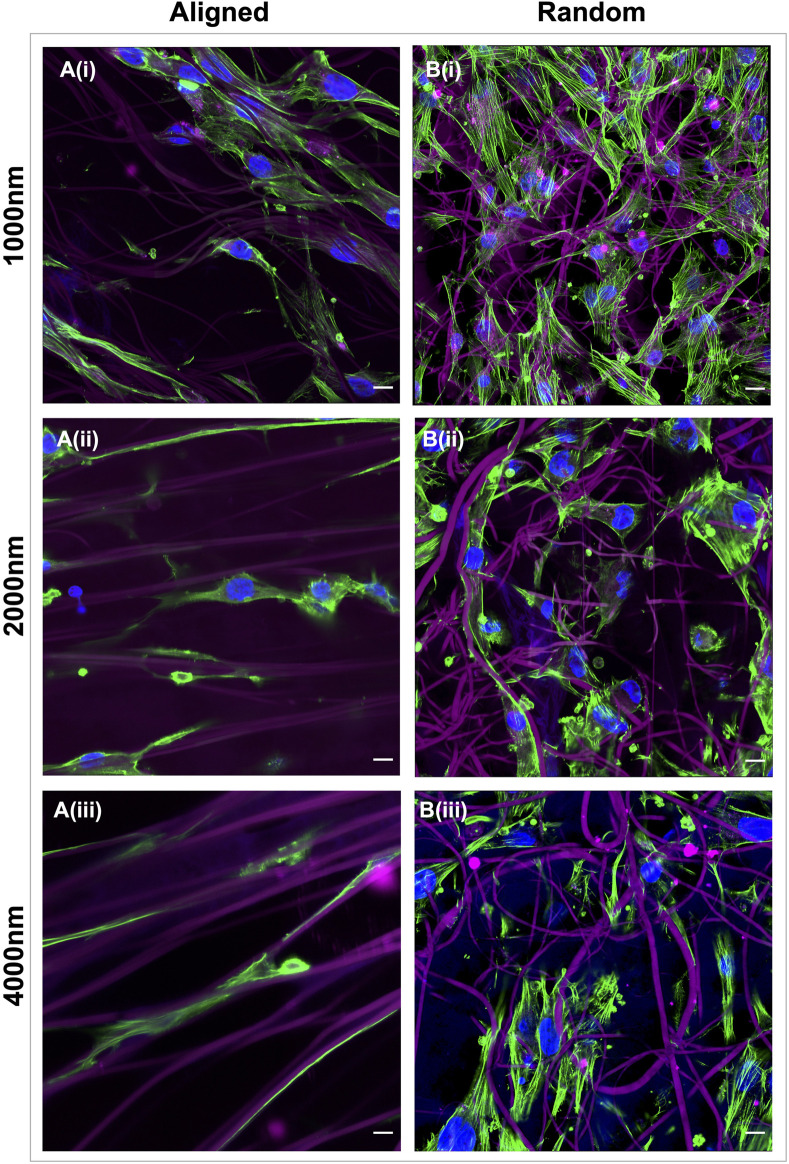
Electrospun scaffold architecture modulates the morphology of healthy tendon fibroblasts. Representative confocal images of healthy hamstring tendon fibroblasts cultured for 7 days on electrospun scaffolds with an aligned **(A)** or random **(B)** orientation of fibers and target fiber diameters of 1000 nm **(i)** 2000 nm **(ii)** or 4000 nm **(iii)**. Green = phalloidin actin stain, Blue = DAPI nuclear stain, Magenta = scaffold auto-fluorescence. Scale bars = 10 µm.

Healthy tendon fibroblasts seeded for 7 days onto scaffolds with aligned fibers demonstrated a distinctive elongated morphology (Figures 3Ai–iii), whilst those cultured on randomly configured fibers demonstrated a flattened and spread morphology (Figures 3Bi–iii). No difference in morphology was observed for fibroblasts cultured on scaffolds with a 300 nm aligned or 300 nm random configuration (Supplementary Figure S2).

### Scaffold Architecture Modulates the Transcriptional Profile of Both Healthy and Diseased Tendon Fibroblasts

**FIGURE 4:**
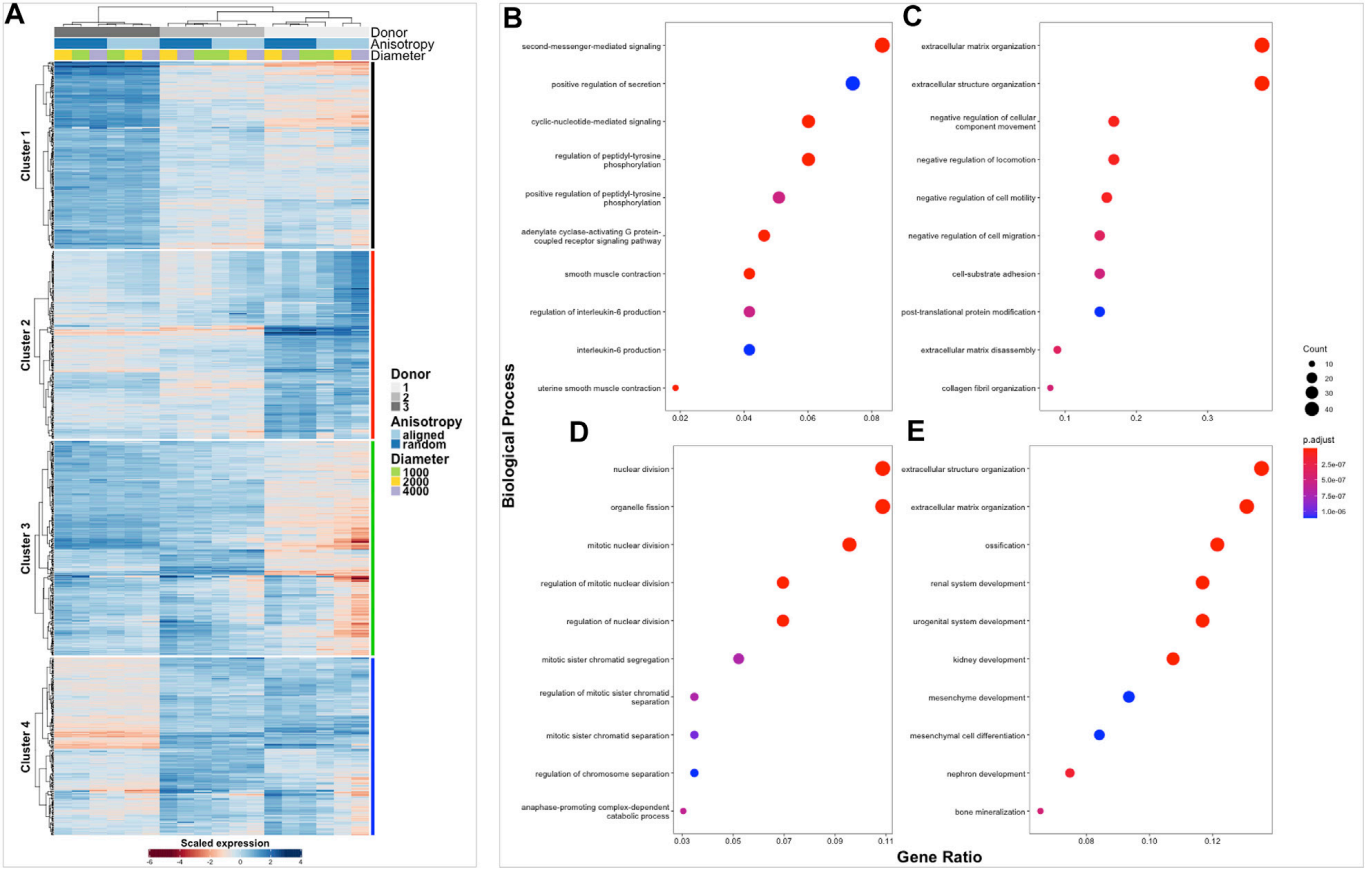
Clustered heatmap of healthy tendon fibroblasts cultured on electrospun scaffolds. The Heatmap depicts 2-way hierarchical clustering of the top 500 PCA genes across all 24 RNA samples sequenced from healthy human fibroblasts seeded on to 6 different electrospun scaffolds. Independent clustering of the samples according to donor, anisotropy and diameter is depicted by the dendrogram **(A)**. Gene clusters are functionally annotated by over representation analysis of gene ontology (GO) biological processes **(B–E)**.

**FIGURE 5:**
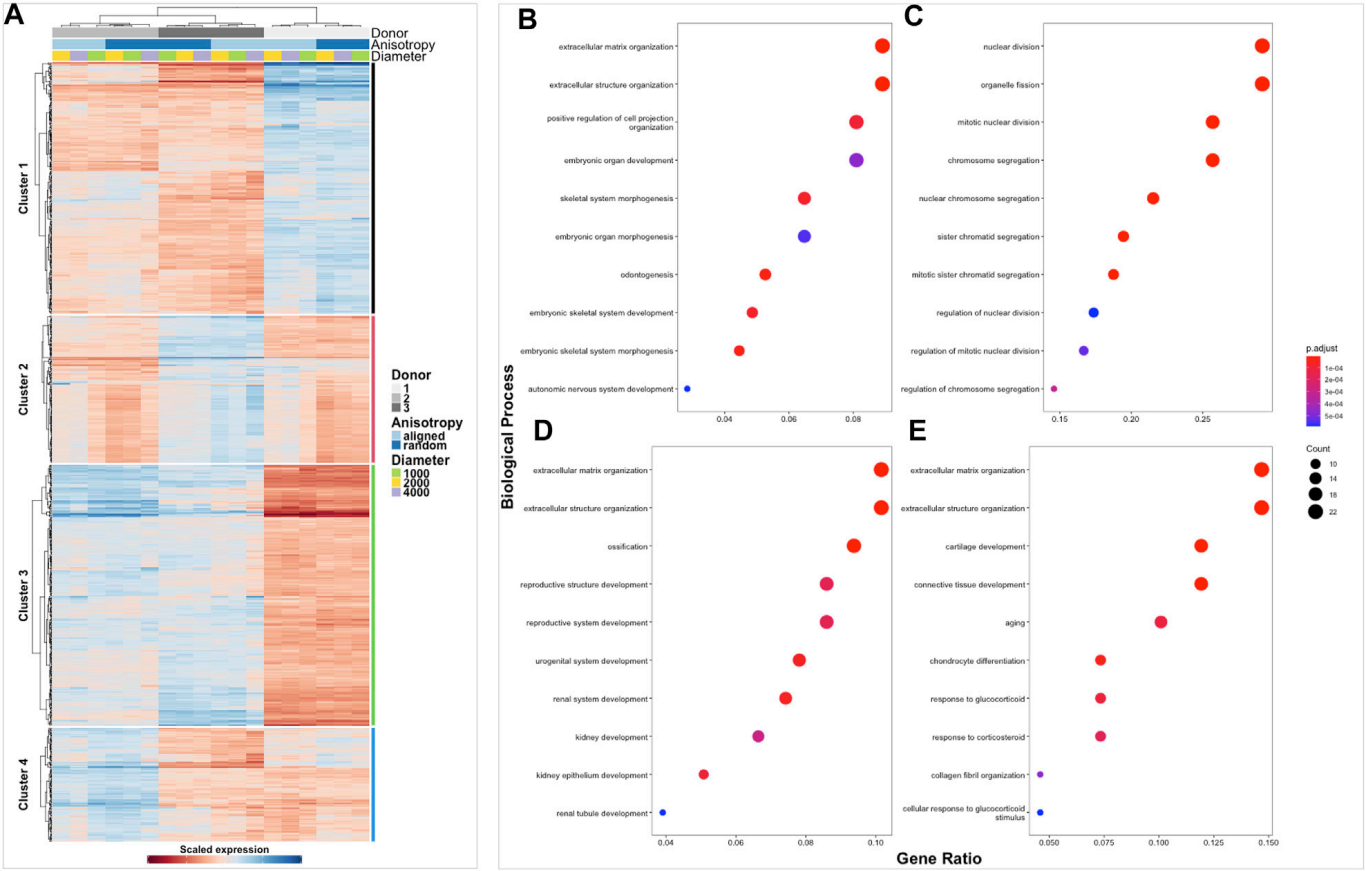
Clustered heatmap of diseased tendon fibroblasts cultured on electrospun scaffolds. The Heatmap depicts 2-way hierarchical clustering of the top 500 PCA genes across all 24 RNA samples sequenced from diseased human fibroblasts seeded on to 6 different electrospun scaffolds. Independent clustering of the samples according to donor, anisotropy and diameter is depicted by the dendrogram **(A)**. Gene clusters are functionally annotated by over representation analysis of gene ontology (GO) biological processes **(B–E)**.

The transcriptome of healthy hamstring (*n* = 3) and massive rotator cuff tear (*n* = 3) derived fibroblasts, cultured on the six distinct electrospun scaffolds (1000 nm, 2000 nm, 4000 nm aligned and random) for 7 days, was assessed using bulk mRNA sequencing. Principle component analysis demonstrated that the largest Euclidean distance was displayed between the different biological donors, PC1 accounting for 70% of the variance in hamstring samples, whilst PC2 represented 21% and seemed to reflect scaffold anisotropy (Supplementary figure S3A). Comparison of PC3 with PC4 also allowed the effect of scaffold anisotropy on gene expression to be more easily visualised. Diseased tendon fibroblasts also responded to scaffold microarchitecture (Supplementary figure S3B). A similar pattern was observed with hierarchical clustering analysis (Figures 4A, 5A) with scaffold-based culture of healthy and diseased tendon fibroblasts influencing three main biological themes; cell cycle regulation, extracellular matrix organisation and tissue maturation (Figures 4B–D; Figures 5B–D).

### Effect of Fiber Anisotropy on the Transcriptome of Healthy and Diseased Tendon Fibroblasts

**FIGURE 6:**
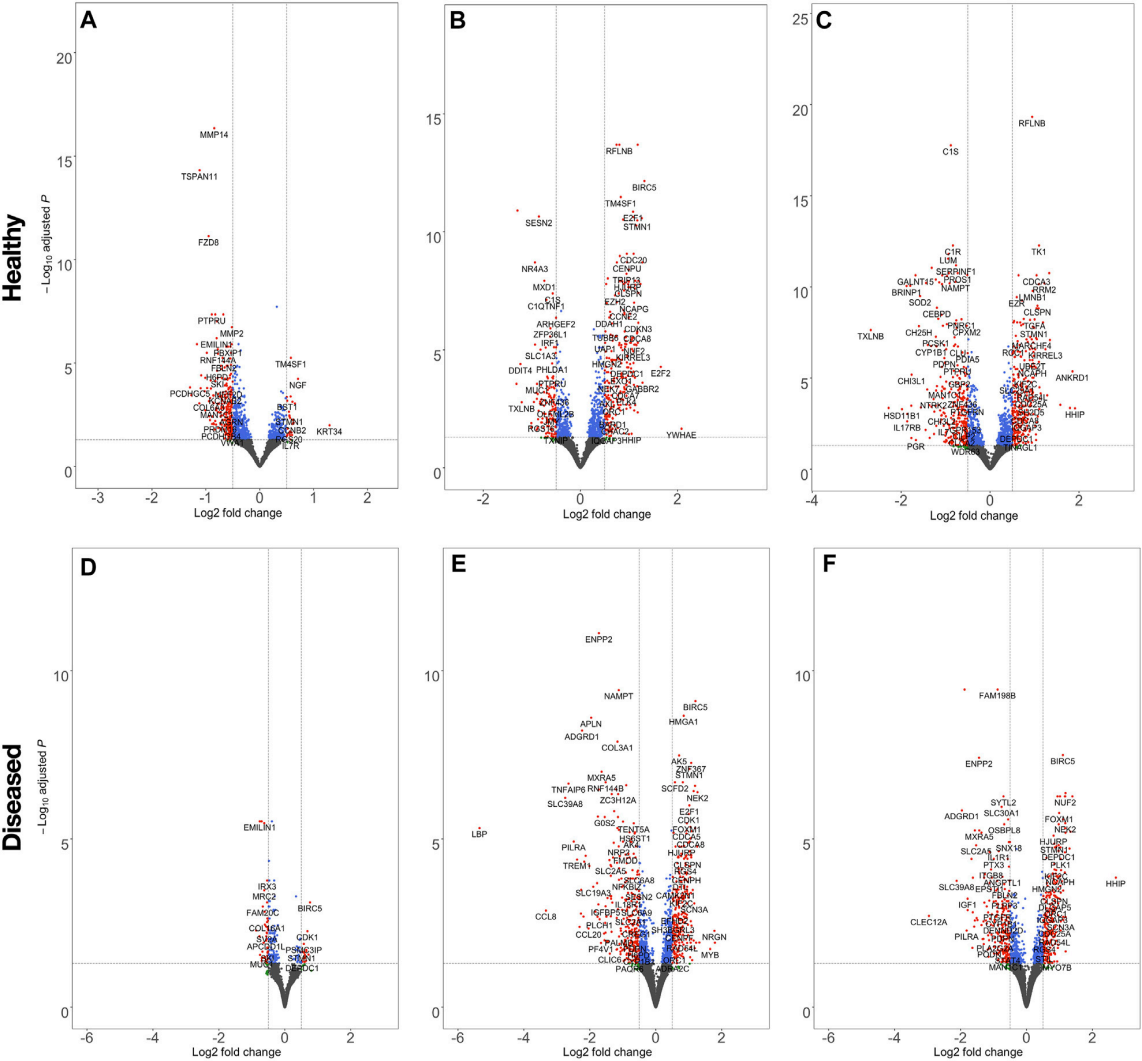
Volcano plots highlighting the effect of scaffold anisotropy on healthy or diseased tendon fibroblast gene expression. Pairwise analysis of scaffolds, seeded with healthy **(A,B,C)** or diseased **(D,E,F)** tendon fibroblasts, with aligned fiber directionality compared with random orientations, but with constant mean fiber diameters of 1000 nm **(A,E)**, 2000 nm (B,E), 4000 nm **(C,F)**. Dashed lines denote a false discovery rate (FDR) of 0.05 and a log2 Fold Change ± 0.5. Transcripts coloured red have passed the thresholds for FDR and log2 Fold-change criteria, blue only meet the FDR, green only the log2 Fold-change threshold, whilst for grey neither criteria are met.

**FIGURE 7:**
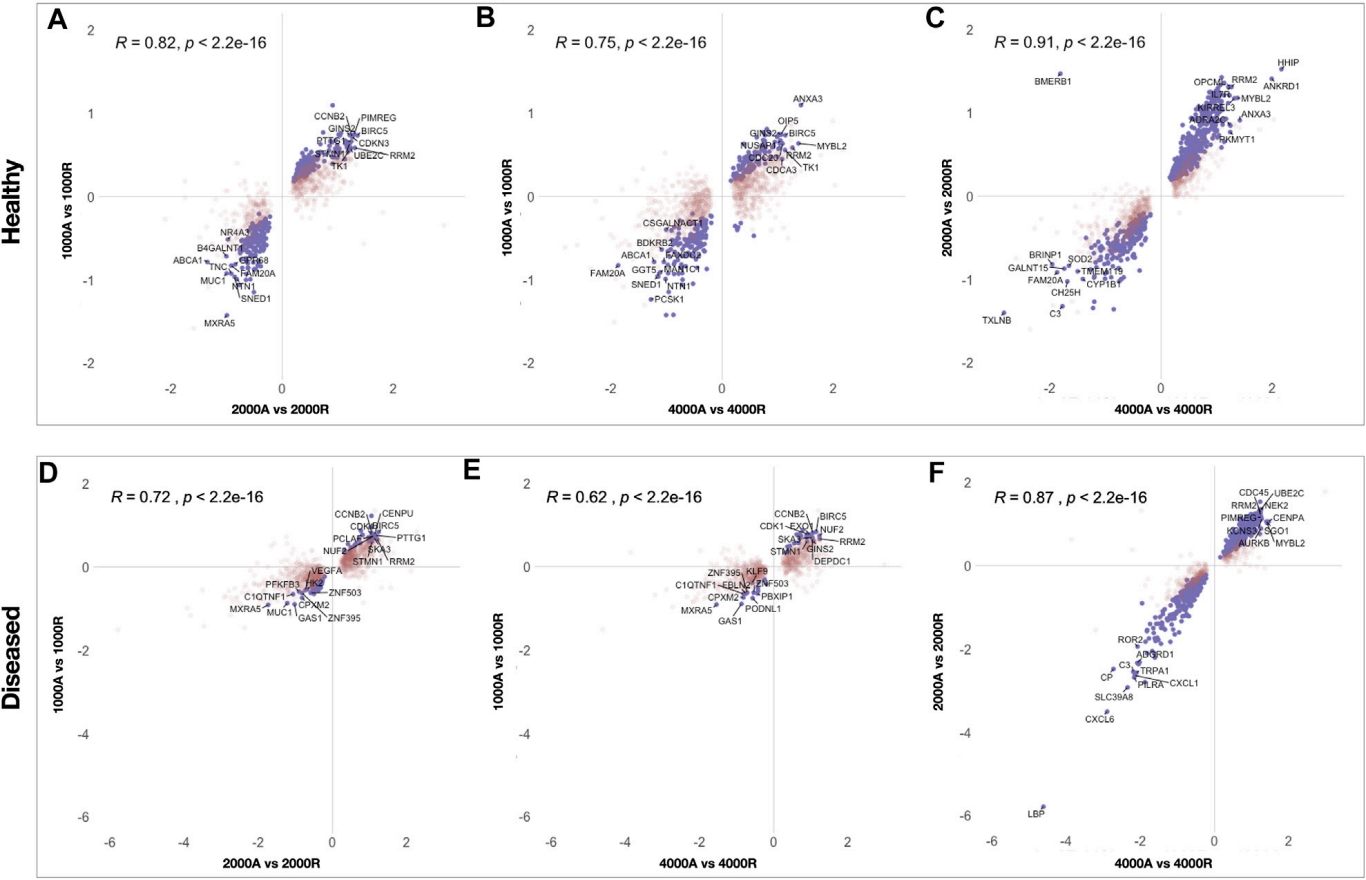
Scatterplots showing correlation of differentially expressed genes between pairwise comparisons of electrospun scaffolds. To identify similarities between pairwise comparisons the Log2FC of differentially expressed genes, for each comparison, were plotted for healthy **(** **A**, **B**, **C** **)** and diseased **(** **D**, **E**, **F** **)** fibroblasts. Genes that reached significance (FDR <0.05) in both comparisons are coloured purple whilst genes in red reached significance in only one comparison. Pearson correlation (r) was used to test the linear dependency of comparisons.

The effect of fiber anisotropy on the transcriptome of diseased tendon fibroblasts was examined across three distinct mean fiber diameters (1000 nm, 2000 and 4000 nm). Pairwise comparisons were made between scaffolds with statistically comparable mean fiber diameters, but with aligned or random fiber configurations. Fiber directionality for aligned scaffolds with 300 nm fiber diameters (Figure 1A iii) demonstrated greater alignment heterogenicity and were excluded from further transcriptional analysis. For both healthy and diseased tendon fibroblast, anisotropy had a marked effect for large diameter (2000 and 4000 nm) scaffolds but had a limited effect on gene expression for scaffolds with a mean fiber diameter of 1000 nm (Figure 6). Differentially expressed genes on 2000 nm aligned *vs*. 2000 nm random scaffolds strongly correlated with differentially expressed genes observed for 4000 nm aligned *vs*. 4000 nm random, suggesting that larger diameter aligned scaffolds have a shared biological effect (Figure 7).

### Large Diameter Aligned Scaffold Influence Fibroblast Activation

**FIGURE 8:**
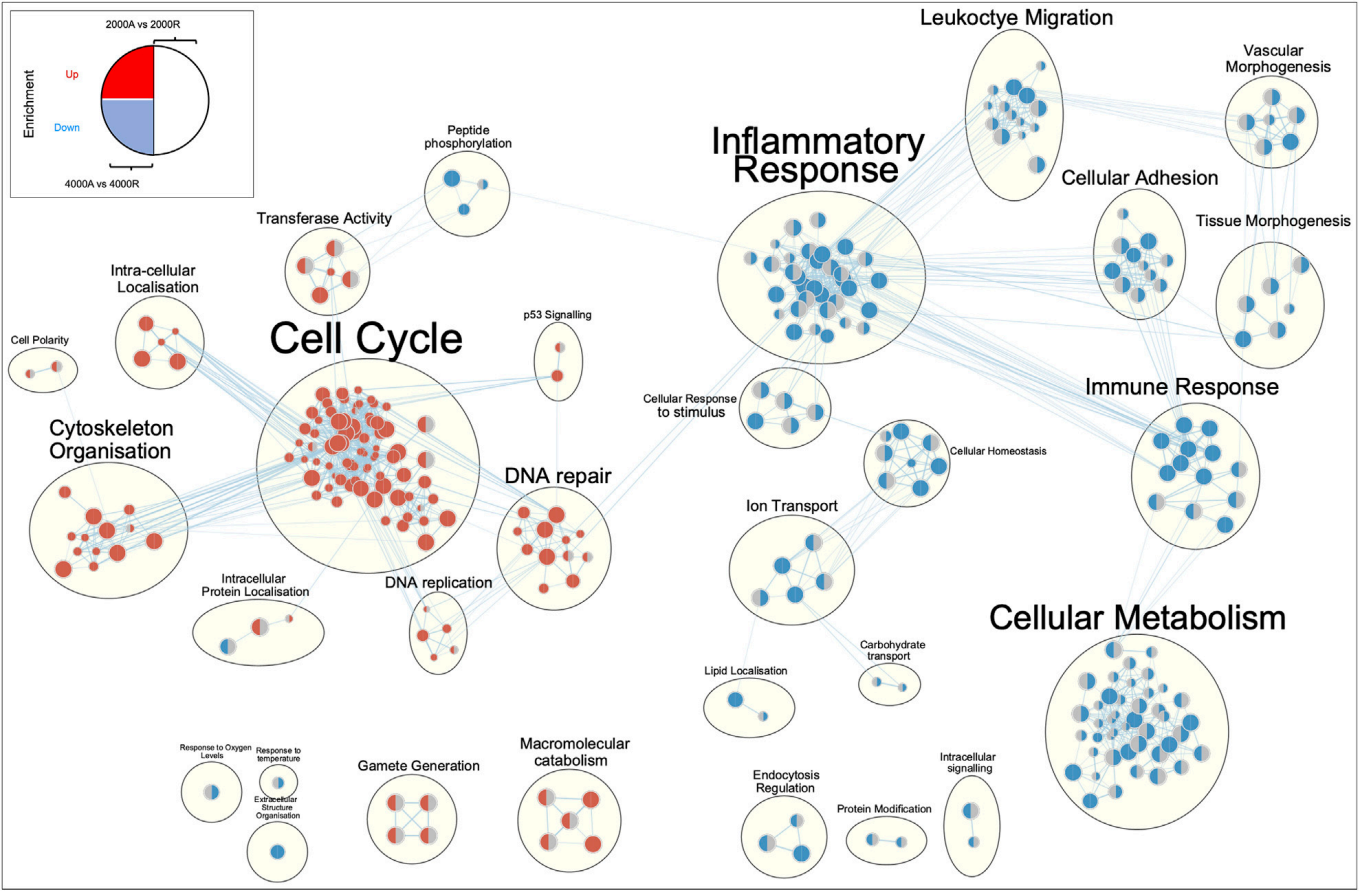
Enrichment map of differentially expressed genes for diseased tendon fibroblast cultured on 2000 nm or 4000 nm aligned *vs*. randomly orientated electrospun scaffolds. Gene set enrichment results were mapped as a network of gene sets (nodes) related by mutual overlap (edges), where the colour (red or blue) indicates direction of regulation. The left-hand side of each node represents enrichment results for 4000 nm aligned *vs*. 4000 nm random comparison, whilst the right-hand side 2000 nm aligned *vs*. 2000 nm random. A grey semi-circle indicates that gene set was not enriched for a particular comparison. Node size is proportional to the total number of genes in each set and edge thickness represents the number of overlapping genes between sets.

**FIGURE 9:**
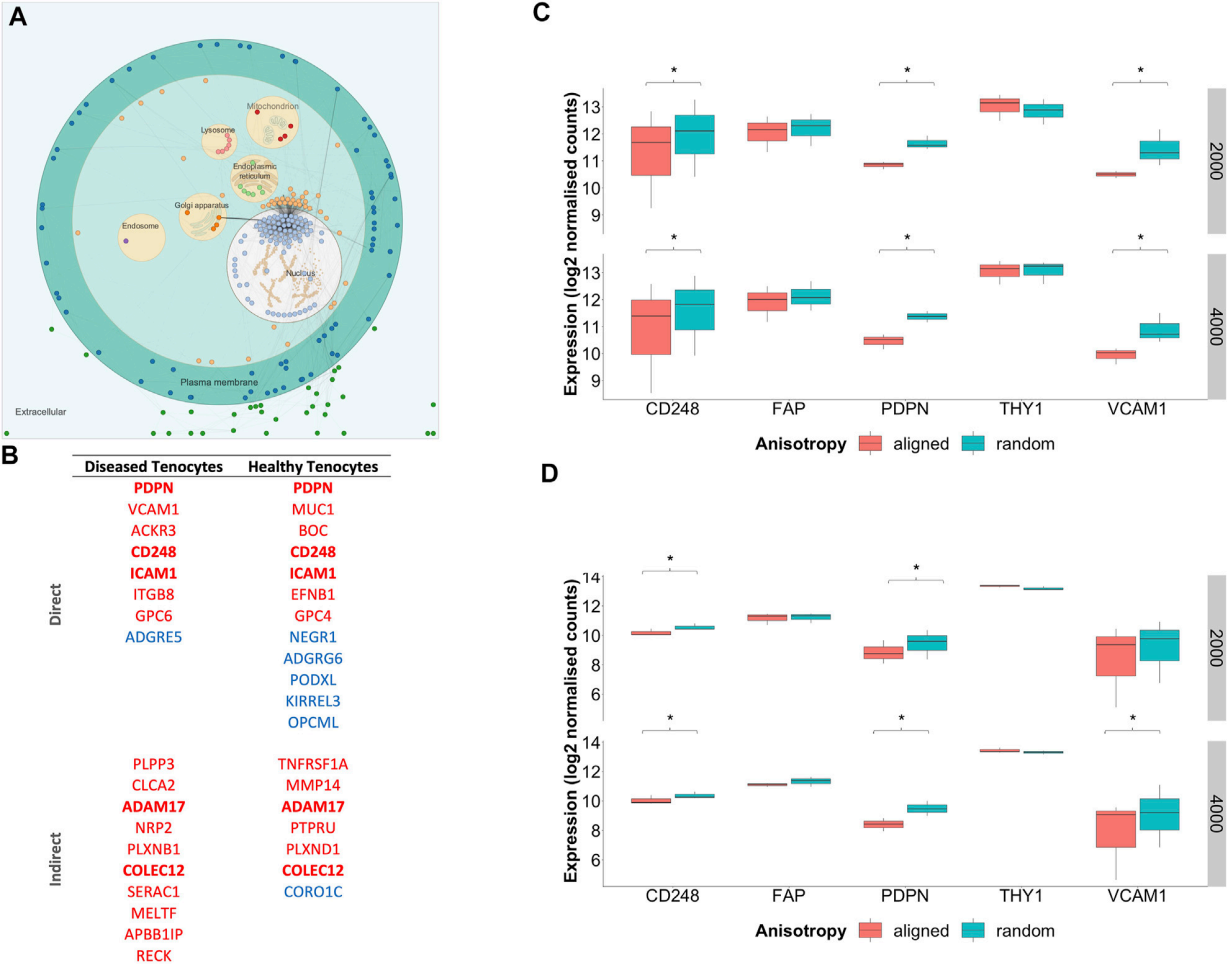
Subcellular location of scaffold regulated genes and expression of fibroblast activation markers. **(A)** Visualisation of commonly differentially regulated genes for diseased tendon fibroblasts cultured on aligned vs random scaffolds of both 2000 and 4000 nm diameters. Nodes represent genes whilst edges demonstrate known functional interactions **(B)** Genes were filtered for those whose translated protein is both localised within the plasma membrane and has a known cell-adhesion and/or extracellular matrix binding function. Genes highlighted in bold represent those that displayed common differential expression across diseased and healthy tendon fibroblasts cultured on aligned *vs*. random scaffolds of both 2000 and 4000 nm diameters. Red indicates downregulation and blue upregulation with genes divided into those with a direct or in-direct function on cell-adhesion. **(C)** Expression of fibroblast activation markers for diseased tendon fibroblasts cultured on PDO scaffolds with aligned or random configuration with 2000 nm or 4000 nm fiber diameter. **(D)** Expression of fibroblast activation markers for healthy tendon fibroblasts cultured on PDO scaffolds. *Denotes p-adjusted (Benjamini-Hochberg correction) value of <0.05. Expression values represent normalised count data transformed to a log2 scale.

To profile the biological processes involved in the transcriptional response of diseased fibroblasts to large diameter (≥2000 nm) aligned scaffolds, gene set enrichment analysis (GSEA) was performed. To simplify interpretation, redundant biological pathways were collapsed into single biological themes and visualised as an enrichment map (Figure 8). Large diameter aligned scaffolds induced an upregulation of genes involved in DNA and cellular replication and a downregulation of genes defining inflammatory responses and cell adhesion. We next examined which genes might be responsible for driving this observed scaffold-fibroblast response. Differentially regulated genes were filtered for those whose translated protein is both localised within the plasma membrane and has a known cell-adhesion or extracellular matrix binding function. Interestingly, this included the fibroblast activation markers CD248, PDPN and VCAM1, suggesting they may serve an important role in fibroblast response to scaffold architecture (Figure 9). In comparison to randomly orientated scaffolds, fibroblast activation marker expression was downregulated, for both diseased and healthy tendon fibroblasts, during culture on aligned scaffolds with large (≥2000 nm) fiber diameters (Figures 9C–D).

## Discussion

This study provides new insights into the effects of microarchitecture on the global transcriptome of tendon derived fibroblasts. We identify that large diameter (≥2000 nm) aligned scaffolds induce a spindle shaped morphology, a relative reduction in gene sets describing inflammatory response, as well as a relative reduction in the transcriptional expression of the fibroblast activation markers CD248 and PDPN. To date, the effect of substrate topology on the gene expression of fibroblasts has undergone only a superficial interrogation, with the current literature predominately focused on assays of proliferation or migration. Indeed, a previous study examining the effect of anisotropy on the expression of 84 cytoskeletal genes is by far the most comprehensive transcriptional profiling otherwise undertaken. (Fee et al., 2016).

Fiber diameter of ES materials is a commonly manipulated variable, with a general trend in the tissue engineering field towards investigating successively smaller nanofibers. Improvements in NIH3T3 fibroblast adhesion (Chen et al., 2007), increased dermal fibroblast proliferation (Hodgkinson et al., 2014) and modulation of stem cell differentiation into ligament-like cells (Bashur et al., 2009) have been reported for sub-micron scaffolds and may have spurred this trend. However, several groups have suggested a critical diameter of 1000–2000 nm, with a reduction in proliferation observed for diameters either side of this range. (Kumbar et al., 2008; Liu et al., 2009; Hsia et al., 2011). Appreciable differences in fiber alignment between all of these studies may help to explain the discrepancy. Therefore, to robustly investigate the effect of fiber diameter we produced scaffolds with three different diameters in either random or highly aligned configurations, enabling any diameter-anisotropy interactions to be interrogated. While at fiber diameters of 1000 nm changes in anisotropy had a limited transcriptional effect, more marked responses to alignment were observed at 2000 and 4000 nm, with shared responses at these diameters suggesting a common biological mechanism. Whilst we did not experimentally interrogate the cellular mechanisms responsible for these biomaterial-cell interactions we observed differential expression of several cell-adhesion related genes. This response appeared conserved across both diseased and healthy fibroblasts cultured on large diameter (≥2000 nm) aligned scaffolds. Similarly, Liu et al. demonstrated a diameter dependent reduction in focal adhesions, with a more chaotic organisation of cell-surface integrins observed for human dermal fibroblasts cultured on submicron electrospun poly (methyl methacrylate) fibers. (Liu et al., 2009).

The terms fibroblast activation, cancer-associated fibroblasts (CAFs) and myofibroblasts all describe an overlapping process by which fibroblasts produce cytokines, chemokines, extracellular matrix proteins and facilitate immunomodulation. (Kalluri, 2016). Activated fibroblasts are a fundamental feature of acute wound healing but their persistence has also been described in malignancy as well numerous musculoskeletal diseases, including tendinopathies. Fibroblasts isolated from rotator cuff tears demonstrate increased expression of the activation markers *PDPN*, *VCAM1* and *CD248*. (Dakin et al., 2017b). There is a long-accepted correlation between fibroblast activation and cell shape (Kalluri, 2016). Indeed, the bilateral relationship between cellular morphology and phenotype is a central tenet of tissue engineering. Electrospun scaffolds with variable anisotropy have previously been shown to be able to manipulate the morphology and differentiation of human progenitor stem cells. (Zhang et al., 2015; Rowland et al., 2016). This is the first exploration of whether the induction of morphological changes can modulate the activation status of adult human fibroblasts. Whilst we cannot exclude that our observations are a transient phenomenon, it is tempting to speculate that scaffold induced changes in cell-shape facilitates fibroblast reprogramming. For murine tendon cells this biophysical reprogramming appears to be driven through epigenetic mechanisms. (Zhang et al., 2018). Since epigenetic dysregulation has also been implicated in persistent fibroblast activation (Klein et al., 2012), future work should interrogate the epigenetic status of tendon fibroblasts in response to scaffold architecture.

There are several limitations to this work. Next generation technologies can provide a powerful insight into the global effect of biomaterials on complex cellular systems. Nonetheless, conventional bulk sequencing can only provide an average expression signal across all cells present, with the assumption of homogeneous cell populations critical to its validity. The phenotype of explanted tendon cells is not currently well characterised, raising the possibility that multiple populations of stromal cells, such as fibroblasts, endothelial cells or pericytes, are present during culture. Subpopulations of fibroblasts, with distinct functions have been described for synovial derived fibroblasts (Croft et al., 2019), and may also exist in tendons. (Kendal et al., 2020). Indeed, distinct functions for epitenon derived and tendon core fibroblasts have already been described in murine and equine models. (Dyment et al., 2013; Cadby et al., 2014). We cannot exclude that a scaffold mediated biasing of stromal cell populations is responsible for the observed transcriptional shifts, an area that single-cell RNA sequencing will be critical to unravelling.

Secondly, we have presented an analysis of cell-material interactions after 7 days of *in vitro* culture. This transcriptional cross-section may explain why tendon fibroblasts, cultured on ≥1000 nm aligned scaffolds, exhibited a downregulation of activation markers and inflammatory pathways, but had not yet also displayed other classical features of cellular quiescence, namely a reduced expression of cell-cycle genes. Alternatively, we cannot exclude that the observed transcriptional changes are a transient phenomenon. Indeed, the matching of material properties to the requirements of each stage of tendon healing will require a temporal understanding of cell-material interactions and remains the focus of future work. Additionally, since we have not provided a transcriptional assessment of cells at baseline (Passage 0) we cannot exclude that short-term cell culture activates tendon fibroblasts, which in turn become primed to ECM or architectural cues. Transcriptional profiling of fibroblasts during prolonged cell culture would also enable the interaction of phenotypic drift and biomaterial architecture on fibroblast activation to be dissected.

Thirdly, fibroblast activation describes a cell-state with recognised changes in the expression of a limited number of surface proteins, including VCAM-1 and PDPN. However, these are surrogate markers of an inflammatory phenotype and may not have a direct functional effect on pathogenic activity. (Crowe et al., 2021). To this end, with the advent of next-generation sequencing, cell-state transitions which typically involve hundreds or even thousands of genes, may be better described at the whole transcriptome level. We present an assessment of activation status at the transcriptional level but have not confirmed whether this translates into a change in stromal cell phenotype (e.g. cytokine secretion) which remains the focus of future work.

Lastly, the generalisability of our findings is currently unclear. We present data generated from a single set of manufactured scaffolds, raising the possibility that unintentional variations in material properties, for example stiffness, total polymer mass or surface chemistry, are responsible for the effects this study has attributed to fiber diameter and anisotropy. We also demonstrating a strong linear correlation between pore area and fiber diameter, with aligned scaffolds demonstrating significantly larger pore sizes than their randomly orientated counterparts. This presents a confounding variable and future work must separate the effect of fiber alignment and pore size on tendon fibroblast phenotype, including the expression of cell-adhesion receptors. For example, the utilisation of sacrificial fibers would enable the manufacture of scaffolds with matched anisotropy and fiber diameter but adjustable pore size. Furthermore, we have not explored the effect of scaffold architecture on cell density, which may confound the observed association between scaffold induced changes in cell shape and transcriptional response. Further detailed mechanistic work is needed before any causal relationship between anisotropy or fiber diameter and fibroblast activation is assigned.

## Conclusion

We have shown for the first time that, at a transcriptional level, the activation of tendon derived fibroblasts is modulated by the anisotropy of large diameter electrospun scaffold fibers. It appears that disordered scaffolds, resembling the architecture of type III collagen that is typically present during the earlier phases of wound healing, resulted in tendon fibroblast activation. Conversely, scaffolds mimicking aligned larger diameter collagen I fibrils, present during tissue remodelling and in healthy tendon, induce a more quiescent phenotype. This has implications for the design of scaffolds used during rotator cuff repair augmentation. These must avoid inducing persistent stromal activation, a characteristic feature of failed tendon healing, and instead provide tissue-resident fibroblasts with temporally appropriate signals to drive successful tendon repair.

## Data Availability

The original contributions presented in the study are publicly available. This data can be found here: https://www.ncbi.nlm.nih.gov/geo/query/acc.cgi?acc=GSE190085
