# Supplementary figures and images for: Electrospun Scaffold Micro-Architecture Induces an Activated Transcriptional Phenotype within Tendon Fibroblasts
Source: Front Bioeng Biotechnol. 2022 Jan 12;9:795748. doi: 10.3389/fbioe.2021.795748 (PMC8790033; doi:10.3389/fbioe.2021.795748)

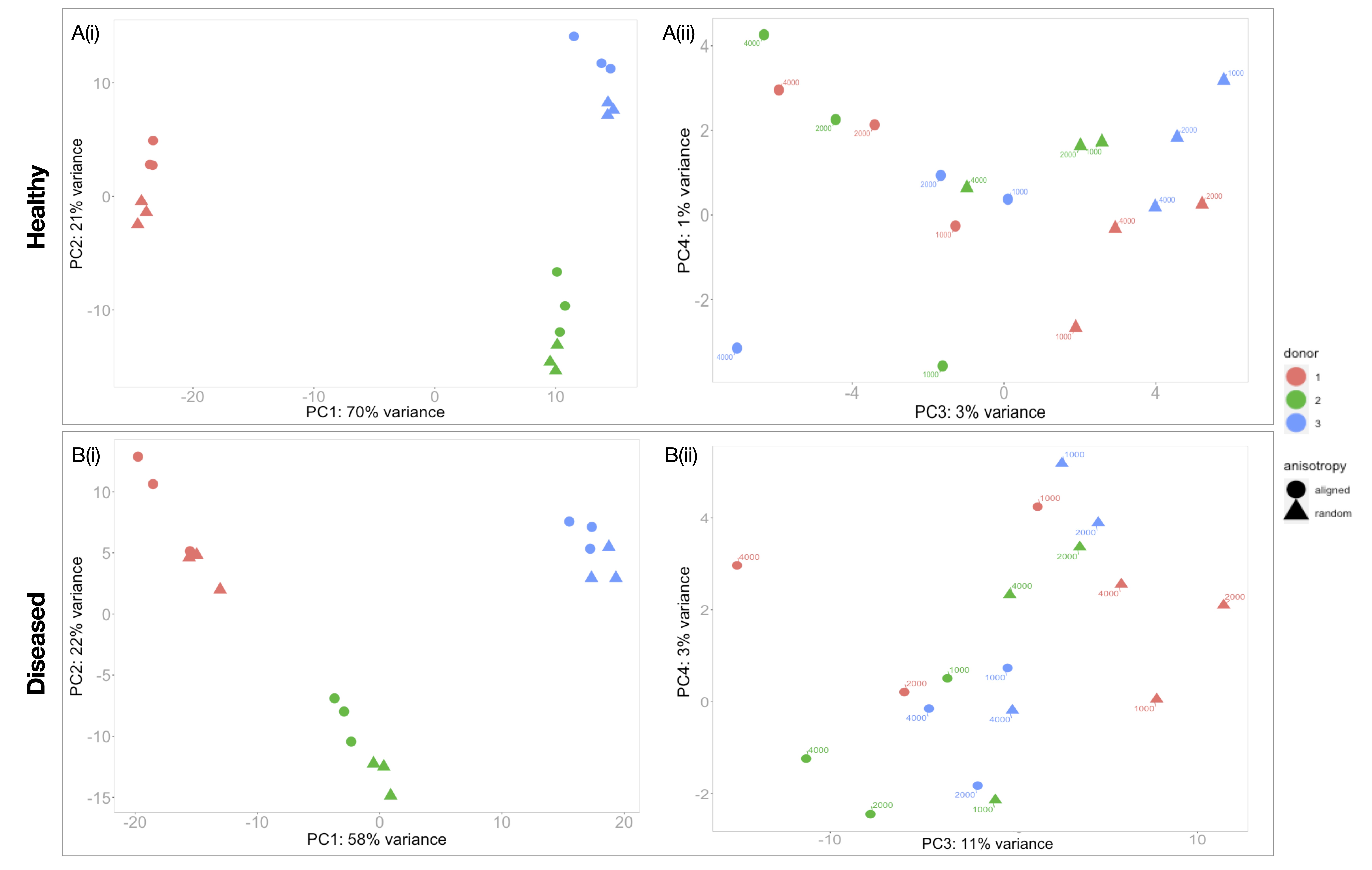

Supplement: Supplementary file 1 [file Image3.JPEG]

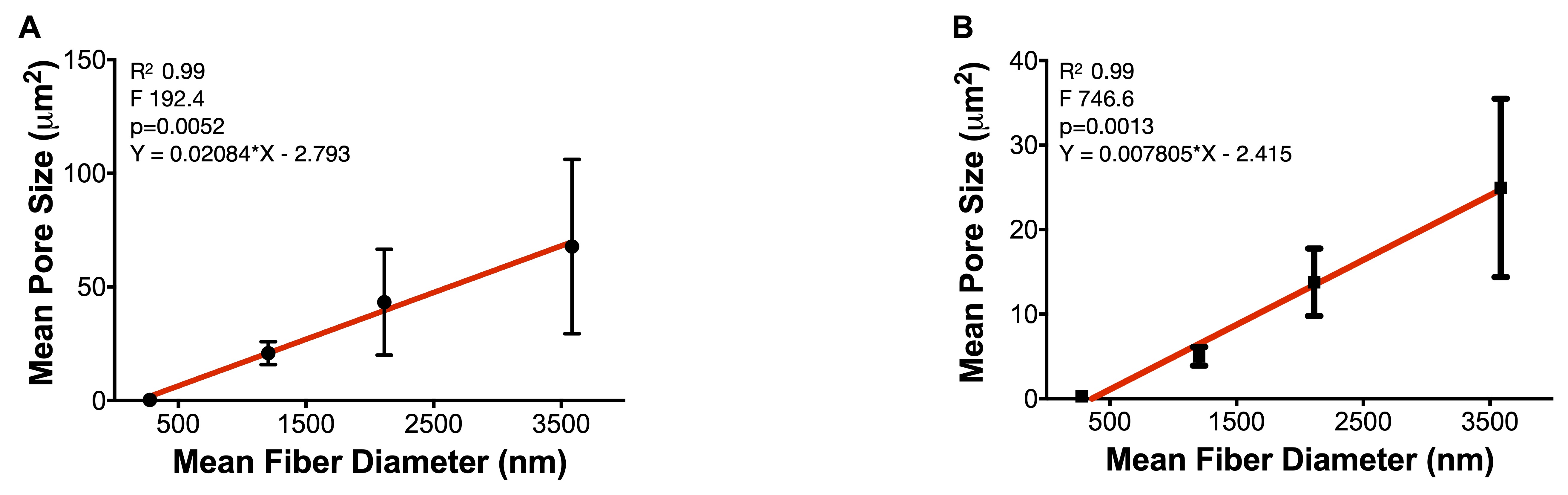

Supplement: Supplementary file 2 [file Image1.JPEG]

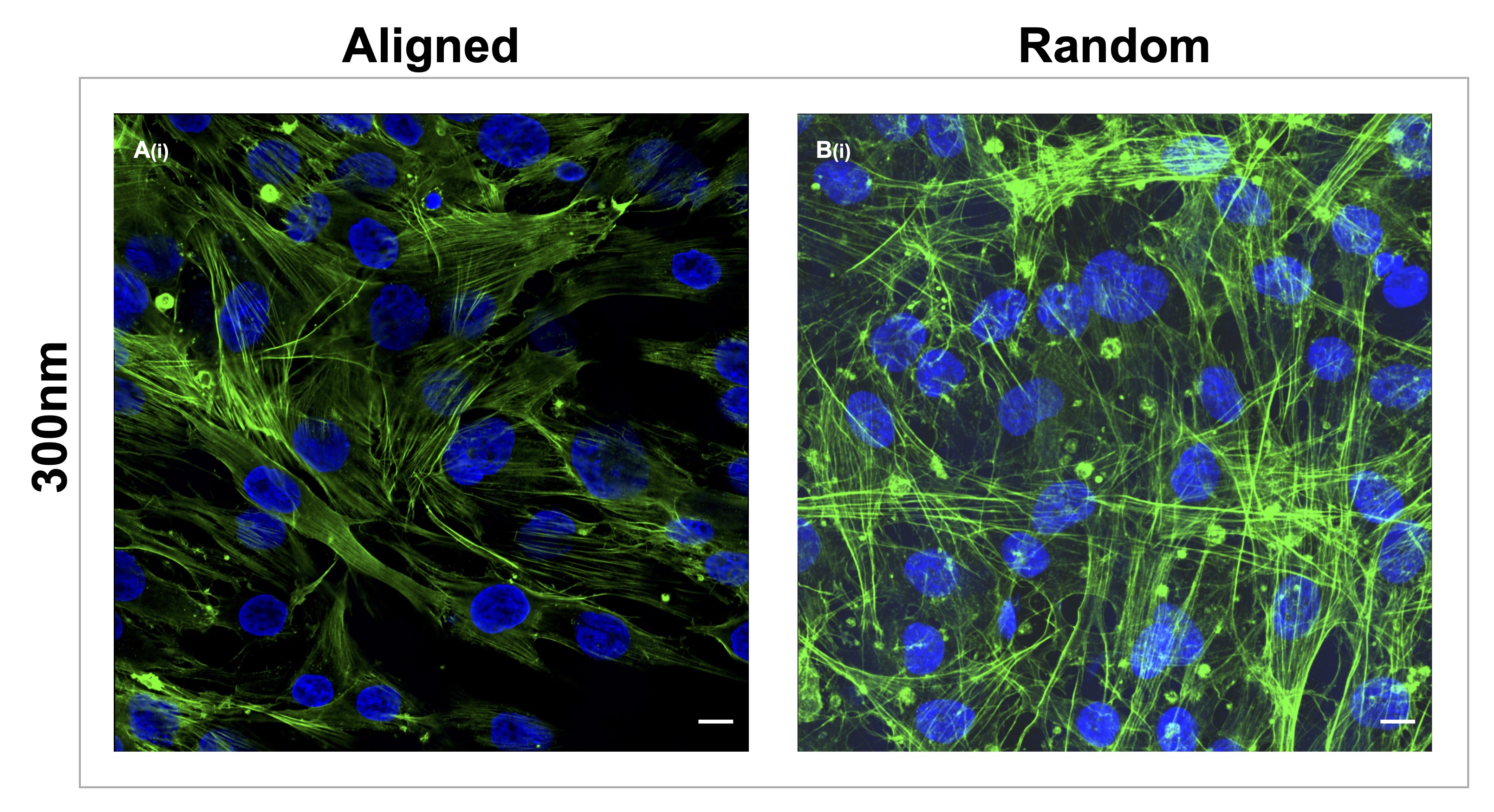

Supplement: Supplementary file 3 [file Image2.JPEG]
