# Supplementary material for: Electrospun Scaffold Micro-Architecture Induces an Activated Transcriptional Phenotype within Tendon Fibroblasts
Source: Front Bioeng Biotechnol. 2022 Jan 12;9:795748. doi: 10.3389/fbioe.2021.795748 (PMC8790033; doi:10.3389/fbioe.2021.795748)
Supplement: Supplementary file 4 [file DataSheet1.docx]

**Supplementary Table 1.** Parameters utilised to produce aligned electrospun scaffolds for *in vitro* culture

| **Scaffold** | **300nm**  **aligned** | **1000nm aligned** | **2000nm aligned** | **4000nm aligned** |
| --- | --- | --- | --- | --- |
| **PDO concentration (%w/v)** | 7 | 12 | 14 | 15 |
| **Pyridine Concentration (μl/ml)** | 1 | 0 | 0 | 0 |
| **Flow rate (ml/hr)** | 0.5 | 0.5 | 1.5 | 3 |
| **Voltage (kv)** | 20 | 8 | 12 | 6 |
| **Collector Speed (rpm)** | 2000 | 2000 | 2000 | 1500 |
| **Nozzle-collector distance (cm)** | 20 | 20 | 20 | 20 |
| **Total time (hours)** | 4 | 4 | 4 | 3 |
|  | | | | |

**Supplementary Table 2.** Parameters utilised to produce random electrospun scaffolds for *in vitro* culture

| **Scaffold** | **300nm**  **random** | **1000nm random** | **2000nm random** | **4000nm random** |
| --- | --- | --- | --- | --- |
| **PDO concentration (%w/v)** | 7 | 10 | 10 | 13 |
| **Pyridine Concentration (μl/ml)** | 1 | 1 | 0 | 0 |
| **Flow rate (ml/hr)** | 0.5 | 0.5 | 0.5 | 0.75 |
| **Voltage (kv)** | 25 | 8 | 10 | 8 |
| **Collector Speed (rpm)** | 50 | 50 | 50 | 50 |
| **Nozzle-collector distance (cm)** | 20 | 20 | 20 | 20 |
| **Total time (hours)** | 4 | 4 | 4 | 3 |
|  | | | | |
